# Authentic Leadership, Personality Types, and Job Satisfaction: Study with Nurses in a Private Hospital

**DOI:** 10.1155/2023/6656568

**Published:** 2023-11-03

**Authors:** Paula Hirayama, Paula Vitali Miclos, Olga Guilhermina Dias Farah

**Affiliations:** Faculdade Israelita de Ciencias da Saúde Albert Einstein, Sao Paulo, Sao Paulo, Brazil

## Abstract

**Objective:**

To identify the authentic leadership dimensions demonstrated by nurse leaders and to analyze the relationship between these dimensions, personality types, and job satisfaction among both leaders and their followers.

**Methods:**

A descriptive, cross-sectional study was conducted with 111 nurses using the Instruments Authentic Leadership Questionnaire, Job Satisfaction Survey, and Myers–Briggs Type Indicator®. The statistical methods used included Spearman correlation coefficients, nonparametric Mann–Whitney tests, and generalized estimating equation models with gamma distribution.

**Results:**

There was a significant difference between the evaluations of authentic leadership by leaders and followers. There was a significant association between authentic leadership and job satisfaction among followers (*p* value >0.05), while no correlation was observed between authentic leadership, job satisfaction, and personality type among leaders.

**Conclusions:**

The findings indicate a positive correlation between authentic leadership dimensions and job satisfaction among followers.

## 1. Introduction

The competitive global environment, characterized by market fluctuations and frequent financial crises, pushes organizations to reinvent their work style [1]. This applies to the hospital environment, which is constantly shaped by technological advancements, market competitiveness, and customer demands. Consequently, the need to modify work practices becomes essential, and leadership is crucial in guiding teams and promoting the development of behavioral skills to achieve improved outcomes [1, 2].

Among the various leadership models identified in theories developed by scholars, authentic leadership (AL) has emerged in the last decade, with an approach centered on transparency, information sharing, and ethical conduct. Characterized by the dimensions of self-awareness, transparency, balanced processing, and moral and ethical perspective, the authentic leader exerts great influence over followers, listening to their opinions, involving them in decision-making processes, actively empowering and developing the team, and earning respect and recognition, which has a direct influence on job satisfaction [1]. Authenticity in a leadership role has a positive and significant relationship with psychological empowerment, contributing to organizational citizenship behavior, fostering a better work environment, promoting team development [3], and positively influencing job satisfaction [4, 5].

Among the various leadership models, AL is not yet formally known by many professionals in the health area; however, it can be identified in different dimensions within hospital organizations, both in the care area and in senior management [6, 7]. Research in the field of nursing has shown that managers and coordinators can, through their authenticity, promote greater learning in their subordinates [7, 8]. Authenticity in the leadership role has a positive and significant relationship with psychological empowerment, increasing the behavior of organizational citizenship, promoting a better work environment and team development [3], in addition to positively influencing job satisfaction [4, 5]. By presenting their “self” in an authentic and transparent way, the leader shows who they truly are, what they believe in, and what their values and goals are, creating bonds and intimacy with their team [9, 10].

As leadership is a significant factor in organizational performance, it is necessary to identify the factors that influence leaders [3]. This can be observed through personality types, which reveal individual characteristics and can serve as a guiding factor and a relevant tool for the development of leadership [11–13].

The Nursing Now Campaign, launched in 2018 by the International Council of Nurses, the World Health Organization, and the UK All-Party Parliamentary Group on Global Health, emphasized the importance and need for recognizing nurses in leadership roles. One of its objectives was to train nursing professionals with a focus on leadership and provide them with more opportunities through increased investment in education and improved working conditions [14]. In addition to this movement, the COVID-19 pandemic has further highlighted the importance of nurses as leaders in patient care, decision-making, and crisis management [15]. Therefore, this study aimed to identify the dimensions of authentic leadership among nurse leaders in a private hospital and examine the relationship between these dimensions, personality types, and job satisfaction among both leaders and their followers.

## 2. Materials and Methods

### 2.1. Design

This is a descriptive, cross-sectional, nonexperimental, quantitative study guided by the STROBE tool. It was conducted from November 2020 to March 2021 in a large private hospital located in the city of Sao Paulo, Brazil. The hospital comprises neonatal, adult, pediatric, oncology, and maternity units, as well as an emergency department, an intensive care unit, and a diagnostic and preventive medicine unit.

### 2.2. Ethical Considerations

The study was approved by the research ethics committee of the institution studied (CAAE: SGPP: 3966-19) and complied with Resolution 466/12 of the National Health Council, which addresses research involving human beings [16].

The participants were informed that their personal information and responses would be anonymized to ensure research confidentiality. They were also informed of their right to withdraw from the study at any time.

Written consent was obtained from those who voluntarily agreed to participate through the signing of the informed consent form (TCLE).

### 2.3. Setting and Sample

The population was chosen for the study by purposive sampling, since it was necessary to be composed of nurses with specific positions. The study population consisted of nurses, divided into two categories: leaders, characterized by the position of senior nurse, and followers, composed of junior or mid-level nurses. The nurses worked in various areas, including medical-surgical units, oncology, emergency care, critical care, outpatient clinics, and diagnostic medicine. In the institution studied, there are three types of nursing leadership roles directly associated with patient care: the senior nurse, who is a professional directly involved in the training of team members and actively participates in the decision-making process related to nursing practices as well as conflict management, together with the area coordinator. The senior nurse is considered a nurse leader and is in constant development to assume the next leadership positions, such as, for example, nursing coordinator. The second leadership role is that of the nursing coordinator, who is responsible for transmitting institutional strategies and policies to the nursing team, monitoring local indicators, and ensuring the correct sizing of employees. Finally, the last leadership position is the nursing manager, responsible for all hospitalization units assigned to him, with managerial functions more focused on decision-making and always aligned with the institution's strategy. In addition to the leadership roles, in the institution under study, there are junior and mid-level nurses, considered in the study as followers. The junior nurse has the characteristic of being a newly graduated and less experienced professional, with the role more focused on bedside care with the patient. On the other hand, mid-level nurses, because they have more experience, are responsible for managing patient care and performing more complex activities than junior nurses. Both junior and mid-level nurses report directly to senior nurses.

The inclusion criteria for both leaders and follower nurses were having a permanent position in the department and at least 6 months of experience in their current role and area of work. This timeframe was considered sufficient for nurses to have knowledge of their role within the team, as well as familiarity with routines, practices, policies, and the work team. Leaders were required to have junior and/or mid-level nurses under their technical responsibility and to have completed the Myers–Briggs Type Indicator® (MBTI®) questionnaire, which assesses personality types through questions related to behaviors in specific situations [17, 18]. Nurses on vacation, medical leave, or maternity leave were excluded from the sample.

Out of the 56 leaders invited to participate in the research, 30 signed the informed consent form and completed the questionnaires provided. However, among them, 6 leaders did not receive corresponding responses from their followers. As it was necessary to link the leader's response with, at least the response of one follower, these 6 leaders were excluded. Thus, the final sample of leaders consisted of 24 senior nurses. A sample size of 24 leader nurses is sufficient to test a correlation coefficient of 0.50 with a power of 64%, considering a significance level of 5% and a Spearman correlation coefficient, calculated by the PASS software [19].

Out of the 260 eligible followers, 87 (33.4%) signed the informed consent form and completed the questionnaires provided. These participants constitute the final sample of followers.

### 2.4. Instruments

#### 2.4.1. Sociodemographic Questionnaire

The questionnaire was developed by the authors and included questions related to sociodemographic characteristics, academic background, years of experience as a nurse, years of experience in the studied hospital, shift worked, and, for senior nurses, the number of junior and/or mid-level nurses under their responsibility.

#### 2.4.2. Authentic Leadership Questionnaire

The Authentic Leadership Questionnaire (ALQ) measures the four dimensions of authentic leadership: transparency, self-awareness, balanced processing, and moral and ethical perspective [20, 21]. The ALQ has been translated, adapted, and validated in Portuguese and consists of 16 items [22]. Scores on the ALQ range from zero to 64, and respondents use a 4-point Likert scale, where 0 corresponds to “never” and 4 to “frequently, if not always.” Higher scores indicate greater authenticity. In this study, the SELF version of the ALQ was used for leaders' self-assessment, while the RATER version was used for the evaluation of leaders by their followers [20, 21]. The use of the ALQ requires purchase and permission. Permission to use the two questionnaires was granted by Mind Garden at no cost through an online request to use the tool for research purposes [21].

#### 2.4.3. Job Satisfaction Survey

The Job Satisfaction Survey (JSS) is an instrument developed by Paul E. Spector to measure job satisfaction. The Portuguese version of the survey, translated, adapted, and validated, consists of 32 items [9, 23]. The questionnaire is divided into nine domains: pay, promotion, supervision, fringe benefits, contingent rewards, operating conditions, coworkers, nature of work, and communication [9, 23]. Participants rate each item on a 6-point Likert scale, where 1 represents “strongly disagree” and 6 represents “strongly agree.” The total scale score can range from 32 to 192, with higher scores indicating greater job satisfaction [9, 23].

#### 2.4.4. Myers–Briggs Type Indicator®

Created by Myers and Briggs, the Myers–Briggs Type Indicator® (MBTI®), the inventory was the result of the authors' research based on Jung's Theory of Psychological Types. Created by Myers and Briggs, the Myers–Briggs Type Indicator® (MBTI®), the inventory was the result of the authors' research based on Jung's Theory of Psychological Types. According to Jung, through personality types, it becomes possible to understand the relationships between “oneself” and the “other” in a constructive way [12, 13, 17, 24]. In addition to psychic attitudes, Jung realized that the psyche has four basic functions, which are distinguished from the other functions, which are sensation and intuition, classified as functions of perception or irrational, and thought and feeling, these functions being judgmental or rational. The pairs of psychic attitudes, as well as the introvert versus extrovert attitude, are opposite to each other and also complementary, with each type being either introverted or extroverted [13, 17]. Myers and Briggs, based on Jung's study, added a subdivision to the existing typology. Defined as judgment and perception, the two typologies refer to the way the individual chooses a perceptive or judgmental attitude towards the way of living, acting, and dealing with the world [17, 24]. One of the results of the research done by Briggs and Myers was the creation and validation of the personality inventory called MBTI®. It aims to use Jung's theory of psychological types in an understandable and useful way, adding the two personality types described by the authors [24]. The Myers–Briggs Type Indicator® (MBTI®) is developed by Myers and Briggs as a contribution to Carl Jung's theory of psychological types. The questions that make up this inventory refer to situations/behaviors with two options of answers, to which the respondent must point out the one that best fits his personality and/or attitude towards the situation described. The results reveal combinations of the Jung, Myers, and Briggs dichotomies: extraversion (E), introversion (I), sensing (S), intuition (N), thinking (T), feeling (F), judging (J), and perceiving (P). The questionnaire's outcome indicates preferences among these dichotomies and reveals one of the 16 personality types, which result from the interactions between individual preferences: ENFJ, INFJ, INTJ, ENTJ, ENFP, INFP, INTP, ENTP, ESFP, ISFP, ISTP, ESTP, ESFJ, ISFJ, ISTJ, and ESTJ [16, 17]. The MBTI® has been translated, adapted, and validated for the Portuguese language, and its distribution and application rights in Brazil belong to the company Fellipelli. The dissemination of the instrument's content is not authorized but there was no need to ask permission to use the questionnaire since the studied institution pays for its use and, for that, is authorized by Fellipelli to apply the tool for training programs focused on employee development [17, 18, 25].

### 2.5. Data Collection Protocol

**Figure 1:**
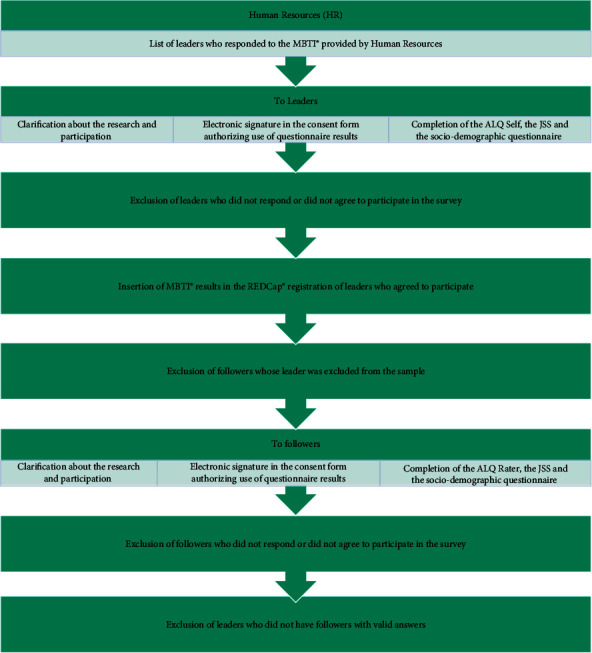
Data collection flowchart.

The data collection process, as depicted in Figure 1, started with the administration of the MBTI® to the nurse leaders by Human Resources employees from the institution, who were qualified to apply the instrument. After that, the names of the leaders and their corresponding results were provided to the researcher. The second stage was the distribution of the research proposal and the sociodemographic questionnaire, the ALQ version SELF, and the JSS via REDCap® for leaders who responded to the MBTI®. In the third stage, nonrespondent leaders and their corresponding followers were excluded, and the MBTI® result was entered into the leaders' REDCap® form, finalizing the sample of leaders. Lastly, the followers were invited to participate in the survey by receiving the sociodemographic questionnaire, the ALQ version RATER, and the JSS, also via REDCap®. To ensure the link between the leader's response and the follower's response, a code was assigned to the leader in the REDCap® tool. This allowed for the insertion of the leader's identifier when registering a follower. Also, when generating reports containing the answers, the results could be analyzed without revealing the identity of the professionals, maintaining the confidentiality of the research.

### 2.6. Data Analysis

The data were described using absolute and relative frequencies for categorical variables. The relationships between authentic leadership scores and job satisfaction of nurse leaders were investigated using Spearman's correlation coefficients. The relationships between the scores in the ALQ, JSS, and MBTI® instruments were evaluated through nonparametric Mann–Whitney tests for each of the 4 aspects, due to the asymmetry observed in the scores [26].

The relationships between authentic leadership scores and job satisfaction of followers were investigated by generalized estimating equation models with Gamma distribution, in order to account for the dependence between the evaluations of different followers regarding the same nurse leader [10]. The analyses were carried out using the software R and SPSS, considering a significance level of 5% [26, 27].

## 3. Results

### 3.1. Profile of Nurses: Sociodemographic Data, Academic Background, and Professional Performance

As presented in Table 1, a total of 111 nurses (24 leaders and 87 followers) participated in the research. The majority of the participants (86.5%; 20 leaders and 76 followers) were female. The mean age of the leaders was 40 years old (31–55 years old), while the mean age of the followers was 36 years old (22–55 years old). Most of the participants (75.7%; 24 leaders and 60 followers) had completed their undergraduate education at least 5 years before to the research. In addition, 74.8% of the participants (21 leaders; 62 followers) had a postgraduate degree in a technical area and 27.9% (11 leaders; 20 followers) in a procedural area. In terms of work performance, 100% [27] of the leaders and 63.2% (55) of followers had been working as nurses for more than 5 years. Moreover, 100% [27] of the leaders and 70.1% (61) of the followers had been working in the hospital studied for more than 5 years. As for time of experience, 41.7% [14] of leaders and 56.3% (49) of the followers had been working in the same position for at least 1 year, while 91.7% [26] leaders and 82.6% (72) of followers had been working in the current area for at least 1 year. Most participants (72.9%; 21 leaders, 60 followers) worked during the day. Most leaders had between 1 and 10 nurses (9; 40.9%, only 22 answered this question) and 31 or more nursing assistants and technicians (12; 50.0%) under their responsibility.

### 3.2. Authentic Leadership and Job Satisfaction in the View of the Leaders and Followers

As presented in Table 2, the mean scores for the dimensions of authentic leadership ranged from 3.3 (transparency) to 3.7 (balanced processing), with a mean total score of 3.4. These results indicate a good self-awareness of the leaders. Conversely, the mean scores among followers were lower when compared to the leaders, ranging from 3.0 for balanced processing and self-awareness to 3.3 for moral and ethical perspective, with an average total score of 3.0.

Regarding job satisfaction, the JSS scores of leaders ranged from 9 to 23 (Table 2), with a mean total score of 104. In the evaluation of followers, the scores per dimension ranged from 9 to 20, with a mean total score of 98.0. The followers' scores were lower than those of the leaders in almost all dimensions. Both leaders and followers obtained higher scores in the supervision dimension and lower scores in the operating conditions dimensions, and both groups were classified as “neither satisfied nor dissatisfied.”

### 3.3. Leaders' Personality Type

The most prevalent personality type among the leaders was ESTJ, accounting for 7 cases (29.2%), followed by ISFJ (5; 20.8%). Two other combinations appeared frequently: ISTJ, with 4 cases (16.7%), and ESTP, with 2 cases (8.3%). Another 6 combinations (ENFJ, ENFP, ENTJ, ESFJ, INTJ, and ISTP) were observed in only 1 leader each (4.16% for each combination).

### 3.4. Correlation between AL and Job Satisfaction for Leaders and Followers

The evaluation of the correlation coefficients indicates an absence or very weak correlation between the dimensions of the ALQ and the total score of the JSS.  Table 3 shows that, although some significant coefficients were observed at the 5% level (*p* value <0.05), highlighted in bold in the table, the values are low, sometimes very close to zero. The highest coefficient is the correlation between the dimension JSS Nature of Work and ALQ Total Score (coefficient = 0.58), but the association is not statistically significant (*p* value = 0.08).

A similar evaluation was conducted for followers, and the coefficients obtained revealed significant and positive correlations. This indicates that a higher perception of authentic leadership is associated with greater job satisfaction. All dimensions of the ALQ, as well as the total score, achieved *p* values below 0.05, which was the significance level adopted in the study when correlating authentic leadership with job satisfaction (all dimensions *p* value >0.001). The moral and ethical perspective dimension stands out, as each one-unit increase in this dimension is associated with a 13.2% increase in the average total score of the JSS (Table 4).

### 3.5. Correlation between AL, Job Satisfaction, and Personality Types of Leaders

To examine the correlation between authentic leadership, job satisfaction, and personality types, the four psychological types were analyzed separately due to the sample size. The analysis of the four typological combinations revealed consistent results across the different types. Even in cases where there are signs of differences between the medians, all *p* values were greater than 0.05 (Table 5).

## 4. Discussion

In the results of this study, the evaluation of participants' perceptions of authentic leadership dimensions using the ALQ revealed higher scores among leaders compared to followers. This finding aligns with a previous Brazilian study conducted with nurses, which explored the correlation between authentic leadership, job satisfaction, and hospital accreditation [4]. Among the leaders, the dimension with the highest score was balanced processing, which indicates their ability to make impartial decisions [20]. Similar findings were reported in a study that examined the impact of a leadership development program focusing on AL behaviors [28]. As for the followers, the highest median score was observed in the self-awareness dimension, a key aspect of AL that reflects how the individual perceives their own influence and meaning in the world based on their knowledge of their personal strengths and weaknesses [20]. Despite the higher median in the self-awareness dimension, the results were very similar across the other dimensions.

As for job satisfaction, leaders had a higher total score on the JSS questionnaire compared to followers. They also presented a higher score in the supervision dimension and a lower score in operating conditions. However, both leaders and followers were classified as “neither dissatisfied nor satisfied,” which is consistent with other studies [4, 29]. Although the classification was the same, the higher score on the JSS questionnaire of the nurse leaders may be related to a clearer knowledge of the career plan, a higher salary, and benefits such as weekend off. These differentials between the leaders and the followers are a characteristic of the hospital under study and may be a justification for this difference in score. A study examining job satisfaction among an oncology nursing team found a positive correlation between job satisfaction and autonomy [30]. Nurses who have control over their practice and the autonomy to make decisions regarding patient care experience greater job satisfaction [8]. Furthermore, higher job satisfaction scores are associated with reduced intention to leave work and lower Burnout syndrome scores [31].

One of the characteristics of the professionals who work in the studied hospital is their autonomy and the support provided by the management in decision-making processes. This demonstrates that nurses are valued and that their freedom in professional practice is recognized, which can directly influence job satisfaction. Moreover, the studied hospital holds the Hospital Magnet® designation, which has as one of its standards a leadership style that supports, advocates for, and empowers staff, with strong nursing leaders at all levels. These factors align well with the authentic leadership model [32].

The Magnet Recognition Program® is an accreditation program administered by the American Nurses Credentialing Center, aimed at improving patient care globally and promoting an environment where nurses, along with the multidisciplinary team, establish and maintain a standard of excellence through leadership, scientific discoveries, and sharing of new knowledge [32]. This demonstrates the importance of supporting leadership in fostering a culture of excellence through engagement and empowerment, ultimately leading to organizational commitment, efficacy, and improved outcomes for both the team and the organization [5, 33–35]. The nurses of the institution under study are encouraged to seek new knowledge, empowerment in care, and increasing involvement with innovation, characteristics that are aligned with the initiatives of the program.

As for the personality types, the most common type observed was ESTJ (7; 29.2%), followed by ISFJ (5; 20.8%). Individuals with an ESTJ MTBI profile are characterized by traits such productivity, responsibility, assertiveness, friendliness, and a realistic approach to decision-making, which align with the characteristics of the authentic leadership dimensions [36]. The predominance of types E and S was also found in an American study carried out with leading nurses with the objective of evaluating the impact of educational modules using the MBTI® tool to understand personality types [11]. In a study that analyzed two samples of leaders, 32q4wone consisting exclusively of physicians and the other comprising a multidisciplinary team, the ISFJ, ESTJ, and ESFJ types were predominant in the multidisciplinary sample, which included nurse leaders [6].

A study conducted in the United States examined the personality types of undergraduate health students participating in a leadership program and found that the use of individual and collective personality typologies in the program helped participants understand their own personality and the personalities of their team members. This understanding led to improvements in the work process and in interpersonal relationships, which are fundamental aspects for the leadership development practices [7].

In the present study, the correlation between the ALQ and JSS scores revealed different results for the two populations. Among the leaders, no statistically significant evidence was found in the association between AL and job satisfaction. However, in the group of followers, a positive and moderate correlation was found in all domains of the ALQ and JSS, except for the association between the total authentic leadership score and satisfaction with operating conditions and the nature of work. Although, in the institution under study, the nursing team has a nursing coordinator, the results demonstrate that the senior nurse has an important role in the development of those followers, influencing, through authentic leadership, job satisfaction. These findings are consistent with a previous study that demonstrated a positive and significant influence of authentic leadership on job satisfaction among followers, while the same correlation was not statistically confirmed among leaders [4]. Furthermore, international publications have also described a positive correlation between AL and job satisfaction dimensions from the perspective of followers [31, 37, 38].

Considering that data collection took place during the challenging period of the COVID-19 pandemic, the results of this study demonstrate the positive impact of AL on the job satisfaction of followers. The perception of authentic leadership among nurses has a negative correlation with frustration related to basic needs and the intention to leave work [35], situations similar to those experienced during the pandemic [39].

Regarding the relationship between AL, job satisfaction, and personality types, no significant correlations were found, except for the predominance of the ESTJ and ISFJ typologies among the leaders. The MBTI® is a valuable tool that professionals can utilize from their training to their self-development journey, aiming to enhance self-awareness [40], which is one of the foundational pillars of AL [21]. The studied institution uses the MBTI® tool for professionals participating in leadership development programs, demonstrating that the institution values this subject, as there is a cost for each evaluation performed [40].

AL has a positive effect on empowerment, which, in turn, has a positive relationship with relational social capital and job satisfaction [31, 41]. Moreover, the literature provides evidence of a positive correlation between AL and the quality and outcomes of patient care, as well as safer attitudes among nursing teams [41, 42]. The authentic leader plays an important role in facilitating the development of followers, promoting learning, creativity, and greater organizational commitment, as well as fostering bonding with the state and personal psychological capital [43, 44] and contributing to the team's innovative behavior [33]. Authentic leaders have a robust ethical and moral character, cultivate transparent relationships, and create trusting environments, exerting influence on the safety climate within the work setting [5, 32, 45].

Job dissatisfaction leads to higher employee turnover and consequently results in a shortage of professionals, which directly impacts patient outcomes and the financial stability of the healthcare system [5, 38]. Authentic leaders, through their ethical behavior, self-awareness, and transparent interactions with their team, strive to foster the growth of their followers, promoting job satisfaction and strengthening organizational commitment [43]. Understanding the personality type of leaders can contribute to the development of AL, as comprehension of this typology can promote self-awareness and lead to improved communication, collaboration, and decision-making processes [37].

### 4.1. Limitations

Our study has some limitations. The first limitation is the use of the MBTI® tool, which can only be applied by a qualified professional, in addition to the cost per application of the inventory, which may have reduced the sample number of leading nurses and, consequently, the number of followers. The second limitation was that the study's collection period coincided with the COVID-19 pandemic. At the time, several questionnaires related to the pandemic survey were sent to the employees of the hospital under study, which may have influenced the number of adherences to the questionnaires. The third limitation is that, due to the stress experienced by the care of patients with COVID during this period, the response of the questionnaires may have been affected by the moment experienced by these nurses.

### 4.2. Implications for Nursing Management

Our results showed that authentic leadership of senior nurses can affect job satisfaction for follower nurses. In this way, the leaders of the units must constantly seek the development of the pillars of authentic leadership, and the MBTI inventory can be an ally in self-knowledge, which is one of the pillars of this leadership style.

## 5. Conclusions

There was a significant difference between the evaluations of authentic leadership of leaders and followers in the dimensions of transparency, self-awareness, balanced processing, and moral and ethical perspectives. The median and total scores were higher in the leader evaluations. Leaders presented higher scores in the balanced processing dimension and smaller scores in the transparency dimension.

Regarding personality types, although certain typologies were more prevalent, there was no significant association between the MBTI®, the ALQ, and the JSS. There was also no association between authentic leadership and job satisfaction among leaders. However, among followers, a positive correlation was found, indicating that a higher perception of authentic leadership was associated with greater job satisfaction, both in terms of overall authentic leadership and its specific domains.

Understanding leadership and the positive impact that leaders can have on their teams is crucial for professional training, and it is essential to conduct studies and develop content in the field of leadership development.

## Figures and Tables

**Table 1:** Demographic characteristics.

	Leader	Followers
(1) Gender		
Female	20 (83.3%)	76 (87.4%)
Male	4 (16.7%)	11 (12.6%)
(2) Age (years)		
Average (standard deviation)	40 (6)	36 (7)
Minimum-maximum	31–55 (18)	22–55 (80)
(3) Time since graduation		
0–5 years	0 (0.0%)	27 (31.0%)
6–12 years	8 (33.3%)	37 (42.6%)
13–20 years	11 (45.9%)	16 (18.4%)
21 years or more	5 (20.8%)	7 (8.0%)
(4) Postgraduate degree in a technical area (such as intensive care, public health, etc.)		
No	3 (12.5%)	25 (28.7%)
Yes	21 (87.5%)	62 (71.3%)
(5) Postgraduate degree in process areas (such as quality, auditing, MBA in management, etc.)		
No	13 (54.2%)	67 (77.0%)
Yes	11 (45.8%)	20 (23.0%)
(6) Years of experience as a nurse		
0–5 years	0 (0.0%)	32 (36.8%)
6–10 years	5 (20.8%)	35 (40.2%)
11–15 years	9 (37.5%)	9 (10.3%)
16 years or more	10 (41.7%)	11 (12.7%)
(7) Years of experience in the current company?		
0–5 years	0 (0.0%)	26 (29.9%)
6–10 years	8 (33.3%)	26 (29.9%)
11–15 years	10 (41.7%)	22 (25.3%)
16 years or more	6 (25.0%)	13 (14.9%)
(8) Years working in the position of nurse supervisor/leader/senior?		
0–6 months	1 (4.2%)	38 (43.7%)
7–12 months	2 (8.3%)	36 (41.4%)
1–5 years	11 (45.8%)	6 (6.9%)
More than 6 years	10 (41.7%)	7 (8.0%)
(9) Nurses under your responsibility^*∗*^		
None	0 (0.0%)	Not applied
1–10	9 (40.9%)	Not applied
11–20	8 (36.4%)	Not applied
21–30	1 (4.5%)	Not applied
31 or more	4 (18.2%)	Not applied
(10) Number of nursing auxiliaries and technicians under your responsibility		
None	0 (0.0%)	Not applied
1–10	3 (12.5%)	Not applied
11–20	6 (25.0%)	Not applied
21–30	3 (12.5%)	Not applied
31 or more	12 (50.0%)	Not applied
(11) Time working in the current area		
0–6 months	2 (8.3%)	10 (11.5%)
7–12 months	0 (0.0%)	5 (5.7%)
1–5 years	4 (16.7%)	37 (42.6%)
6 years or more	18 (75.0%)	35 (40.2%)
(12) Work shift		
Day	21 (87.5%)	60 (69.0%)
Night	3 (12.5%)	27 (31.0%)

^*∗*^22 answers for the 9^th^ question. Leaders = 24; followers = 87.

**Table 2:** Scores of the dimensions of the Authentic Leadership Questionnaire and the Job Satisfaction Survey for leaders (*n* = 24) and followers (*n* = 87), Sao Paulo, 2020.

Questionnaire dimensions	Median (1st; 3rd quartile)	Minimum maximum
*Authentic leadership (ALQ)-SELF leader*		
Transparency	3.3 (3.0; 3.6)	2.4–4.0
Moral and ethical perspective	3.4 (2.9; 3.8)	2.3–4.0
Balanced processing	3.7 (3.2; 3.7)	2.7–4.0
Self-awareness	3.4 (2.9; 3.9)	2.0–4.0
Total	3.4 (3.1; 3.6)	2.6–4.0

*Authentic leadership (ALQ)-RATER follower*		
Transparency	3.2 (2.6; 3.6)	0.4–4.0 (85)
Moral and ethical perspective	3.3 (2.8; 3.8)	0.8–4.0 (85)
Balanced processing	3.0 (2.5; 3.7)	0.7–4.0 (84)
Self-awareness	3.0 (2.3; 3.5)	0.0–4.0 (86)
Total	3.0 (2.6; 3.4)	0.4–4.0 (81)

*Job satisfaction (JSS)-leader*		
Pay (4–24)	16.0 (12.0; 18.5)	8.0–22.0
Promotion (3–18)	14.0 (11.0; 15.0)	7.0–18.0
Supervision (4–24)	23.0 (19.0; 24.0)	14.0–24.0
Fringe benefits (3–18)	12.0 (10.0; 14.0)	3.0–18.0
Contingent rewards (4–24)	17.0 (14.5; 21.0)	9.0–24.0
Operating conditions (3–18)	9.0 (7.5; 10.5)	5.0–13.0
Coworkers (4–24)	19.0 (16.5; 20.0)	15.0–24.0
Nature of work (3–18)	16.0 (15.0; 17.5)	12.0–18.0
Communication (4–24)	19.0 (17.0; 21.0)	14.0–23.0
Total score (32–192)	104.0 (98.5; 120.0)	80.0–130.0

*Job satisfaction (JSS)-follower*		
Pay (4–24)	15.0 (12.0; 17.0)	4.0–24.0 (87)
Promotion (3–18)	11.0 (8.0; 13.0)	3.0–17.0 (87)
Supervision (4–24)	20.0 (17.0; 23.0)	8.0–24.0 (87)
Fringe benefits (3–18)	11.0 (9.0; 13.0)	3.0–18.0 (87)
Contingent rewards (4–24)	16.0 (13.0; 19.0)	4.0–24.0 (87)
Operating conditions (3–18)	9.0 (7.0; 11.0)	4.0–14.0 (87)
Coworkers (4–24)	18.0 (15.0; 20.0)	8.0–24.0 (87)
Nature of work (3–18)	16.0 (14.0; 18.0)	7.0–18.0 (87)
Communication (4–24)	17.0 (15.0; 20.0)	4.0–24.0 (87)
Total score (32–192)	98.0 (88.0; 113.0)	57.0–135.0 (87)

*Note*. ALQ, Authentic Leadership Questionnaire; JSS, Job Satisfaction Survey.

**Table 3:** Spearman correlation coefficients for job satisfaction and authentic leadership scores of leaders (*n* = 24).

	ALQ transparency		ALQ moral and ethical perspective		ALQ balanced processing		ALQ self-awareness		ALQ total score	
	Coefficient	*p*value	Coefficient	*p*value	Coefficient	*p*value	Coefficient	*p*value	Coefficient	*p*value
JSS pay	0.24	0.1	0.22	<0.01	0.26	0.14	0.49	0.39	0.44	0.1
JSS promotion	0.4	0.75	0.27	0.12	−0.07	0.01	0.16	0.03	0.26	0.08
JSS supervision	0.35	0.57	0.33	0.44	0.12	0.3	0.19	0.41	0.35	0.66
JSS finge benefits	0.15	0.02	0.29	0.05	−0.05	0.01	0.03	0.05	0.15	0.01
JSS contingent rewards	0.23	0.1	0.13	0.01	−0.06	0.01	0.18	0.04	0.17	0.02
JSS operating conditions	−0.01	0.03	0.2	0.11	0.13	0.4	0.34	0.95	0.2	0.22
JSS coworkers	0.22	0.02	0.44	0.05	−0.1	<0.01	0.27	0.03	0.3	0.01
JSS nature of work	0.38	0.21	0.6	0.1	0.41	0.26	0.44	0.08	0.58	0.08
JSS communication	0.26	0.46	0.33	0.41	0.06	0.11	0.38	0.69	0.35	0.52

*Note*. ALQ, Authentic Leadership Questionnaire; JSS, Job Satisfaction Survey.

**Table 4:** Relationship between authentic leadership perceived by followers and job satisfaction (JSS total score).

	Ratio of averages (95% CI)	*p* value
Total ALQ	1.138 (1.090; 1.188)	<0.001
ALQ transparency	1.111 (1.061; 1.163)	<0.001
ALQ-moral and ethical perspective	1.132 (1.084; 1.182)	<0.001
ALQ-balanced processing	1.108 (1.060; 1.158)	<0.001
ALQ-self-awareness	1.103 (1.065; 1.142)	<0.001

*Note*. ALQ, Authentic Leadership Questionnaire; JSS, Job Satisfaction Survey.

**Table 5:** Correlation between the dimensions of the Authentic Leadership Questionnaire and the Job Satisfaction Survey for leaders and personality types (*n* = 24), Sao Paulo, 2020.

		Extraversion (*n* = 13)	Introversion (*n* = 11)	*p*value	Sensing (*n* = 20)	Intuition (*n* = 4)	*p*value	Feeling (*n* = 8)	Thinking (*n* = 16)	*p*value	Judging (*n* = 20)	Perceiving (*n* = 4)	*p*value
ALQ transparency	Median (1st; 3rd quartile)	3.2 (3.0; 3.6)	3.4 (2.8; 3.6)	0.733	3.4 (3.0; 3.6)	3.1 (2.7; 3.6)	0.477	3.4 (3.2; 3.7)	3.2 (3.0; 3.6)	0.417	3.2 (3.0; 3.6)	3.6 (3.1; 3.9)	0.431
ALQ moral and ethical perspective	Median (1st; 3rd quartile)	3.5 (3.3; 3.8)	3.3 (2.5; 3.8)	0.150	3.4 (2.9; 3.8)	3.5 (3.0; 3.9)	0.737	3.6 (2.6; 3.9)	3.3 (3.0; 3.8)	0.928	3.5 (3.0; 3.8)	3.0 (2.9; 3.5)	0.575
ALQ balanced processing	Median (1st; 3rd quartile)	3.7 (3.0; 3.7)	3.7 (3.3; 4.0)	0.424	3.7 (3.2; 3.7)	3.7 (3.3; 3.8)	0.682	3.7 (3.2; 4.0)	3.7 (3.2; 3.7)	0.452	3.7 (3.2; 3.7)	3.5 (3.2; 3.8)	0.970
ALQ self-awareness	Median (1st; 3rd quartile)	3.5 (3.0; 3.8)	3.3 (2.8; 4.0)	0.955	3.3 (2.8; 3.9)	3.5 (3.3; 3.8)	0.525	3.8 (3.0; 4.0)	3.1 (2.9; 3.6)	0.192	3.5 (3.0; 3.9)	2.9 (2.6; 3.5)	0.347
Total ALQ	Median (1st; 3rd quartile)	3.4 (3.1; 3.6)	3.4 (2.9; 3.5)	0.569	3.4 (3.1; 3.6)	3.3 (3.1; 3.7)	0.970	3.5 (3.3; 3.7)	3.3 (3.0; 3.5)	0.320	3.4 (3.1; 3.6)	3.2 (3.0; 3.7)	0.575
JSS pay	Median (1st; 3rd quartile)	16.0 (14.0; 18.0)	16.0 (9.0; 19.0)	0.910	16.0 (13.5; 18.5)	11.0 (8.0; 16.5)	0.183	17.5 (10.0; 18.5)	15.5 (13.5; 18.0)	0.881	16.5 (12.5; 19.0)	14.0 (10.5; 15.5)	0.183
JSS promotion	Median (1st; 3rd quartile)	14.0 (12.0; 15.0)	13.0 (11.0; 15.0)	0.691	14.0 (12.5; 15.0)	8.5 (7.0; 12.5)	0.097	15.0 (9.0; 15.0)	13.5 (11.5; 15.0)	0.610	14.0 (11.0; 15.0)	13.5 (10.0; 14.5)	0.682
JSS supervision	Median (1st; 3rd quartile)	24.0 (20.0; 24.0)	22.0 (18.0; 23.0)	0.207	23.0 (20.0; 24.0)	20.0 (17.0; 22.0)	0.309	22.5 (21.0; 23.5)	23.0 (18.0; 24.0)	>0.99	23.0 (18.0; 24.0)	22.5 (21.0; 23.5)	>0.99
JSS fringe benefits	Median (1st; 3rd quartile)	12.0 (8.0; 14.0)	12.0 (11.0; 14.0)	0.331	12.0 (10.0; 14.0)	10.0 (7.0; 12.0)	0.210	11.0 (10.0; 12.0)	12.0 (9.5; 14.0)	0.417	12.0 (10.0; 14.0)	10.0 (7.5; 12.5)	0.309
JSS contingent rewards	Median (1st; 3rd quartile)	16.0 (15.0; 22.0)	17.0 (14.0; 20.0)	0.955	17.0 (15.5; 21.0)	13.5 (11.0; 18.0)	0.157	17.0 (14.5; 19.0)	16.5 (14.5; 22.0)	0.787	17.0 (14.5; 22.0)	16.0 (14.5; 18.0)	0.525
JSS operating conditions	Median (1st; 3rd quartile)	9.0 (8.0; 10.0)	9.0 (7.0; 11.0)	>0.99	9.0 (7.0; 10.0)	10.0 (8.5; 11.5)	0.347	8.0 (7.5; 9.5)	9.0 (7.0; 11.5)	0.417	9.0 (7.5; 11.0)	8.5 (6.5; 9.5)	0.477
JSS collaborators	Median (1st; 3rd quartile)	19.0 (18.0; 22.0)	18.0 (16.0; 20.0)	0.150	19.0 (16.5; 20.0)	18.0 (17.0; 20.0)	0.737	17.5 (16.0; 21.0)	19.0 (17.5; 20.0)	0.610	19.0 (17.0; 21.0)	17.5 (15.5; 19.5)	0.388
JSS nature of work	Median (1st; 3rd quartile)	17.0 (16.0; 17.0)	15.0 (13.0; 18.0)	0.119	16.0 (15.0; 17.0)	17.5 (15.0; 18.0)	0.388	16.0 (15.0; 18.0)	16.0 (15.0; 17.0)	0.834	16.5 (15.0; 17.5)	15.5 (14.5; 17.0)	0.682
JSS communication	Median (1st; 3rd quartile)	19.0 (17.0; 22.0)	19.0 (18.0; 21.0)	0.865	19.0 (18.0; 21.0)	16.5 (16.0; 20.0)	0.388	19.0 (19.0; 21.0)	18.5 (16.5; 21.5)	0.490	19.0 (18.0; 21.5)	16.5 (15.0; 18.5)	0.097
JSS total score	Median (1st; 3rd quartile)	104.0 (100.0; 123.0)	109.0 (93.0; 117.0)	0.910	105.5 (100.5; 121.0)	93.5 (85.5; 109.5)	0.097	106.5 (95.5; 120.0)	104.0 (99.5; 118.0)	0.928	105.5 (98.5; 124.0)	102.5 (96.0; 106.5)	0.431

*Note*. ALQ, Authentic Leadership Questionnaire; JSS, Job Satisfaction Survey.

## Data Availability

The data supporting the study findings are available from the corresponding author upon reasonable request.
